# Telerehabilitation in the Middle East North Africa Region: A Structured Review

**DOI:** 10.5195/ijt.2021.6401

**Published:** 2021-12-16

**Authors:** Naif Qasam Aljabri, Kim Bulkeley, Anne Cusick

**Affiliations:** 1 Faculty of Medicine and Health, the University of Sydney, Australia; 2 College of Medical Rehabilitation Sciences, Taibah University, Saudi Arabia

**Keywords:** Middle East, Northern Africa, Rehabilitation, Telerehabilitation

## Abstract

A structured review using the PRISMA guidelines, MeSH keywords and eight health databases was conducted (1990 to March 2021). Telerehabilitation research evidence from the Middle East and North Africa region (MENA) was summarized. Twelve studies from Iran, Israel, Morocco, and Saudi Arabia met inclusion criteria; nearly all had been published within the past five years. Methodological quality was moderate to good in the four randomized controlled trials, five cohort-studies and three cross-section surveys. There were seven intervention studies in cardiovascular, musculoskeletal, neurology or burn rehabilitation and three patient perception and two practitioner perception studies. Narrative synthesis revealed content themes relating to rehabilitation availability and accessibility; patient/practitioner perceptions of telerehabilitation; telerehabilitation to augment traditional services; and barriers to telerehabilitation. Telerehabilitation practice in MENA has been demonstrated as feasible, acceptable to patients, and effective in practitioner-designed cohort specific programs. Practitioners are generally positive but lack experience and need training, enabling technological systems, and policy frameworks.

Rehabilitation is an integral part of health systems, supporting people who have or are at risk of having disability to maximise their ability to engage in everyday activities and fully participate in their life situations (World Health Organization [WHO], 2017; WHO, 2020). Rehabilitation is provided within a continuum of care from hospital care to rehabilitation in the community (Stucki, Cieza & Melvin, 2007). It includes interventions for preventing impairment and deterioration in the acute phase of care and optimization and maintenance of functioning in the post-acute long-term phases of care (Meyer et al., 2011; Stucki, Rinehardt & Grimby, 2007). The “World Report on Disability” identifies unmet rehabilitation needs across the globe (WHO, 2011), there is an increasing demand for rehabilitation (WHO, 2020), and variable status of rehabilitation as an essential health service (WHO, 2018), particularly in the developing world (WHO, 2011, 2017). This is concerning because the majority of people with disability (80%) live in developing countries (WHO, 2011).

One region where rehabilitation access and availability are problematic is the Middle East North Africa region, also known as MENA (Human Rights Watch, 2014). There are 19 countries in this region and all but one (Israel) is classified by the United Nations as being in ‘developing regions’ (United Nations Statistical Division, 2020). According to the classification used by Human Rights Watch (2020), the countries in MENA are: Algeria, Bahrain, Egypt, Iran, Iraq, Israel/Palestine, Jordan, Kuwait, Lebanon, Libya, Mauritania, Morocco/Western Sahara, Oman, Qatar, Saudi Arabia, Syria, Tunisia, the United Arab Emirates, and Yemen. Currently, there are no aggregate disability prevalence data for MENA countries, however, individual country estimates, using various metrics, range up to 20% (Barlev et al., 2015; Thompson, 2017; United Nations Economic and Social Commission for Western Asia, 2018). There is also no comprehensive data regarding the mix of public, private, or not-for-profit disability and/or rehabilitation services available across the MENA region (Benjadid, 2019; Cusick, & Hamed El Sahly, 2018; Rosen, Waitzberg & Merkur, 2015). Historically, rehabilitation has been a low priority (WHO, 2019). Barriers to rehabilitation access and availability in low and middle-income countries include poor availability, access issues such as service location and transport problems, individuals not being aware of the services that do exist, lack of specialised equipment and/or assistive devices, and the cost of services (Bright, Wallace & Kuper, 2018; WHO, 2011; Zahid et al., 2017). Other problems include rehabilitation workforce shortages and limited skills of rehabilitation providers leading to access and quality issues (Benjadid, 2019; Bright et al., 2018; Cusick, & Hamed El Sahly, 2018; WHO, 2011).

Telerehabilitation has been proposed as an alternative to in-person consultation to provide rehabilitation (Laver et al., 2020). Telerehabilitation is the delivery of rehabilitation services using information and communication technologies (Hung & Fong, 2019; McCue, Fairman & Pramuka, 2010; Molini-Avejonas et al., 2015; Nafai, Barlow & Stevens-Nafai, 2017). By using technologies such as telephone, videophone, videoconferencing, webcams, web apps, online networks, virtual reality, and wearable technology solutions (McCue et al., 2010; Molini-Avejonas et al., 2015; Peretti et al., 2017), people needing rehabilitation services can communicate with professionals or rehabilitation teams and receive consultation and interventions. Sites for telerehabilitation services can include homes, clinics, schools, residential care homes, and other community facilities (Piron et al., 2008; Schutte et al., 2012). Using such technology for remote delivery of services can make rehabilitation more accessible and available (Brennan, Mawson & Brownsell, 2009; Peretti et al., 2017), assisting patients to overcome barriers to program participation (Peretti et al., 2017; Tenforde et al., 2017).

In MENA countries where rehabilitation services are limited in number, concentrated in few locations, poorly interconnected, or with challenges in transport infrastructure and limited community facilities, telerehabilitation may provide a service delivery model with the potential to increase access and capacity. Telerehabilitation processes can include patient consultation, assessment, monitoring, intervention, supervision, and education (Brennan et al., 2009, 2011; Sarsak, 2020). The body of evidence for impact, effectiveness, and cost-effectiveness in different diagnostic groups and with different interventions is still emerging, but promising (Agostini et al., 2015; Hung & Fong, 2019; Laver et al., 2020; McIntyre, Robinson & Mayo, 2020; Sarfo et al., 2018; Torsney, 2003). Investigation into the extent of telerehabilitation in MENA is yet to occur, and to date there has been no review of research into this topic, but there are indications of practice interest. Telerehabilitation has, for example, been recommended in MENA, as a rehabilitation delivery approach in Morocco (Nafai et al., 2017), and as an alternative to in-person consultation during the COVID-19 pandemic (Abolghasemian et al., 2020; Qureshi et al., 2021; Ziade et al., 2020).

This emerging practice comes off-the-back of a longstanding interest in technology-enabled remote health service delivery in MENA. The earliest evidence of e-health use in MENA is in the “Handbook of Telemedicine” (Ferrer-Roca & Sosa Ludicissa, 1999), where installation of internet-based consultation capacity is reported in the King Faisal Specialist Hospital in Saudi Arabia (https://www.kfshrc.edu.sa/en/home). More recently, the World Health Organisation Global Surveys on eHealth (WHO, 2010, 2016) show a slow but upward trajectory in e-health services in the region. The second survey (WHO, 2010) identified that Jordan, Bahrain, Algeria, Israel, and Oman were offering e-health services, while in the third survey Morocco and Mauritania were reported to be undertaking testing and review processes prior to e-health implementation (WHO, 2016, 2017). In addition, country-specific initiatives planning for e-health services are underway; Saudi Arabia for example, has developed national strategic plans for e-health (Ministry of Health, 2013). Building on this momentum, this study aims to identify, summarize and synthesize research relating to telerehabilitation in the MENA region.

## METHODS

A structured literature review was implemented. The review identified the research question, developed a search strategy to access relevant sources, performed study selection, extracted and recorded data; and collated and summarised the results. PRISMA (Page et al., 2021) guidelines were used to report the study method and findings.

A search strategy was developed in consultation with a health sciences librarian using a combination of keywords and medical subject headings (MeSH) terms (Table 1). Eight healthcare databases were used: the Allied and Complementary Medicine Database (AMED), the Cumulative Index to Nursing and Allied Health Literature (CINAHL), the Cochrane Database of Systematic Reviews (CDSR), Excerpta Medica Database (EMBASE), the Joanna Briggs Institute (JBI) EBP Database, Medline, Scopus, and Web of Science (WoS). The databases were searched from January 1990 through December 2020 with an update search covering the period January 2021 through March 2021. The full search strategy for each database is presented in the Appendix.

**Table 1:** Search Strategy

(1) Telehealth OR tele-health OR telemedicine OR tele-medicine OR telerehabilitation OR tele-rehabilitation OR ehealth OR e-health OR mobile health OR mhealth **AND**
(2) Rehabilitation OR habilitation **AND**
(3) Middle East OR Bahrain OR Qatar OR United Arab Emirates OR Yemen OR Iran OR Iraq OR Israel OR Jordan OR Kuwait OR Lebanon OR Oman OR Syria OR Saudi Arabia OR Palestine OR North Africa OR Tunisia OR Egypt OR Morocco OR Western Sahara OR Algeria OR Libya OR Mauritania.

*Note.* In the Human Rights Watch list, the following countries are paired names but have been separated here with OR as paired names are not consistently used in controlled vocabulary indexes: Palestine/Israel and Morocco/Western Sahara.

**Figure 1:**
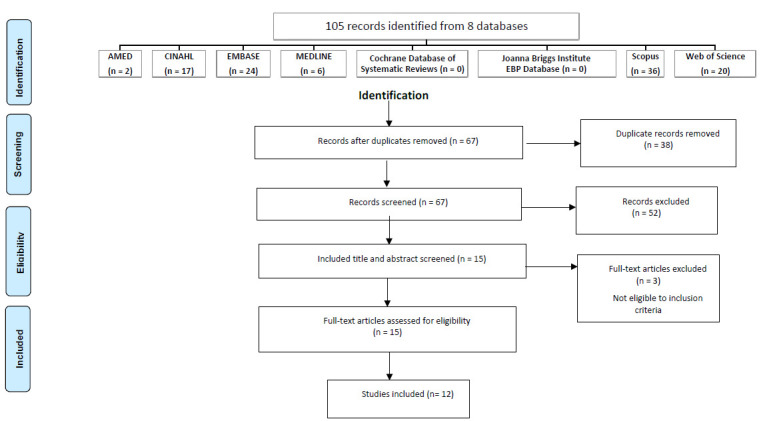
PRISMA Flow Diagram

The first author (NA) located and downloaded all sources into the reference management software Endnote x9™ where identification and removal of any duplicate studies was completed. The data were exported to an Excel™ spreadsheet noting author(s), year of publication, title, abstract, and publication type. The first and last author (NA and AC) independently screened titles and abstracts of all studies to ascertain whether or not inclusion criteria were met (Table 2). It was agreed from the outset that any rating differences would be resolved through consensus-based discussions which involved the second author (KB). A full text version of each screened source (n=15) were independently assessed by NA and AC to determine if inclusion criteria were met. Three papers were excluded following full text review (Figure 1).

**Table 2:** Study Inclusion and Exclusion Criteria

Inclusion criteria	Exclusion criteria
The study collected original data using experimental or observational, survey or qualitative research designs.	Review articles, books, conference abstracts, magazine articles, editorials, perspectives and opinion articles, study protocols, commentaries, policies, guidelines, and reports
An approach was explored that met the definition of telerehabilitation provided by McCue, Fairman & Pramuka (2010): the delivery of rehabilitation services that use a range of information and communication technologies to serve patients, clinicians, and systems.	Any health services that did not meet the definition of telerehabilitation
The study focused on rehabilitation services provided or any medical conditions by at least one of the following rehabilitation professionals: rehabilitation physician, occupational therapist, physiotherapist, speech-language pathologist, psychologist, audiologist, exercise therapist, rehabilitation counsellor or rehabilitation nurse.	The study focused on health services provided by any health professionals who are not indicated as having a focus on rehabilitation.
Original data could be from patients or from rehabilitation professionals.	No original data and/or data that is not from patients or rehabilitation professionals.
The full text study was written in English language.	The full text study was not written in English language.
The study was conducted in MENA countries as classified by the Human Rights Watch.	The study's topics have not specified MENA countries, as classified by Human Rights Watch.
Article was published in a peer-reviewed journal as indicated by (a) Ulrichsweb™ or (b) self-report information from journal's homepage (taken in good faith).	No evidence that the article was published in peer reviewed journal.

The sources (n=12) meeting inclusion criteria had data extracted and entered into an Excel™ spreadsheet by NA and independently cross-checked by AC. Data extracted were: author(s), publication year, title of the study, title of the journal, journal peer review status, study location, study aim, research design used, participant attributes (sample size, age data, gender, whether patients or rehabilitation professionals, types of rehabilitation professionals), name and brief description of intervention (if one was used), outcome measures, type of telerehabilitation platform/modality, telerehabilitation health professional providers and key findings. This data was extracted because individually and cumulatively they characterised what was known about telerehabilitation in MENA in published research.

Three critical appraisal tools were used to assess methodological quality and risk of bias; each matched the study design of included studies. Two reviewers (NA and AC) conducted the appraisals, and consensus decisions were reached regarding each checklist item. To enable aggregate description of methodological quality, the authors developed a cumulative level of attainment for each checklist to indicate whether the paper was poor (< 50% of included checklist items were demonstrated), moderate (50- 80% demonstrated), or good (> 80% demonstrated). The relative attainment for each grade of attainment was modelled on that used in Reilly et al. (2016). Ratings for each study are presented in Table 3. Individual appraisals are available on request. The following critical appraisal tools were used:

- The Critical Appraisal Skills Programme checklist for randomized controlled trials (Critical Appraisal Skills Programme, 2020). All questions for assessing methodological quality were included; two questions relating to the local application were excluded.
- The Critical Appraisal Skills Programme checklist for cohort studies (Critical Appraisal Skills Programme, 2018). All questions for assessing methodological quality were included except number 7 which was excluded because it is a summary question about reported results and did not have a rating scale response. Question number 8 was modified to include the rating scale of ‘Yes, No, and Can't Tell’, the same as questions 1-6. To answer ‘Yes,’ the research needed to report point and interval estimates appropriate for descriptive and inferential results; three questions relating to local application were excluded.
- The Joanna Briggs Institute (JBI) checklist for Studies Reporting Prevalence Data was used for cross-sectional studies (The Joanna Briggs Institute, 2013). This contains nine questions, and all were used.

**Table 3:** Study Characteristics

Source	Year	Country	Research aim	Research design [quality rating]	Sample size Age (mean, variance)	Sample Gender (male n; female n)	Sample primary Diagnosis/ Condition	Measures used in study*	Intervention (brief description)	Disciplines involved in intervention	Technologies used in delivery	Site where rehab received	Outcome
PATIENT INTERVENTION STUDIES													
Alasfour & Almarwani	2020	Saudi Arabia	To determine if the studyspecific Arabic smart phone app ‘My Dear Knee’ increases exercise program adherence.	RCT App group n=20; control group C=20) CASP: Good	N=40 Mean age 54.4 (+/4.33 years)	40 females	Osteoarthritis in knee	Self-reported adherence to prescribed home exercise program; Arabic numeric pain rating scale; Arabic version of the reduced Western Ontario, McMaster Universities Osteoarthritis Index Physical Function subscale, and Five-Times Sit- To-Stand Test scores.	6 weeks exercise program with home exercise - paper resource versus smart-phone-app	Physiotherapist only	Smart phone Author- designed app ‘my dear knee'	home	App group has reduced pain, increased physical function, increased lower limb strength and increased program adherence compared to control
Azma et al	2018	Iran	To evaluate the impact of telerehabilitation office based physiothera py versus conventional therapy on function and OA symptoms.	RCT CASP: Moderate	76 randomized; N=54 completed 58.25 years (+/-7.41; range 45 60 years)	n=21 males n=33 females Reported as a % of all partici-' pants but unclear if this refers to all randomis ed or all completed — completed reported here	Knee osteoarthrit is	Persian version of Knee injury and Osteoarthritis Outcome Score (KOOS); Visual Analogue Scale (VAS); Western Ontario and McMaster Universities Osteoarthritis Index (WOMAC) (pain, AdL, symptom, sport, QoL subscales)	18 sessions/6 weeks each group. Telerehabilitation group received face to face instruction in exercises, information pamphlet, then home practice, with weekly monitoring call by doctor by phone; Control group: passive therapies physiotherapy in clinic services.	Telerehabilitation group: physical therapist instruction then physical rehabilitation specialist doctor follow up; Control group: physical therapist at clinic.	Phone — assisted telerehabilitation call on weekly basis versus physical therapy clinic	Both groups in clinic instruction, then home tele- rehabilit ation group versus in-clinic physiotherapy	Both groups improved on pain and function; no significant difference between telerehabilitation and in-clinic service — telerehabilitation equivalent outcomes to traditional clinic service
Golebowicz et al.	2015	Israel	To determine if an ergonomic intervention followed by electronic biofeedback self-practice for 4–6 weeks at work reduced upper limb musculoskeletal symptoms including pain.	Observational prospective cohort study CASP: Moderate	N=12 34.25 years (2458)	6 males; 6 females	Computer operators with work — related musculoskeletal disorders	Biodemographic questionnaire; Pre-post PROM Standard Nordic Questionnaire; pre-post PROM Swedish Demand Control Support Questionnaire; pre-post Rapid Upper Limb Assessment; surface electromyogra phy; pre-post physical examination for upper extremity symptoms.	Workplace assessment and adjustment; provision of biofeedback and installation of program on each person's workstation; for selfpractice use 4–6 weeks	Occupational therapist only	Exercise data via a telerehabilitation biofeedback system	Workplace	Upper extremity symptoms reduced, regions of pain and activity limitations from pain reduced, body posture improved
Kalron et al.	2018	Israel	To evaluate effects of telerehabilitation on mobility in people following hip surgery.	RCT CASP Good	N=40 randomized; 32 completed mean age controls 67.3 (SD 9.5); telerehabilitation of 65.7. (7.8)	Gender data provided for: 17 males; 19 females	hip surgery	The Timed Up and Go test, 2min walk test, 10-m walk test, sit to stand test, walking speed, and mean step length.	6 weeks, 3 sessions/week telerehabilitation versus control intervention group	Physiotherapist	video-based telerehabilitation program.	home	Telerehabilitation (n=15) significantly higher in all mobility outcomes than control (n=17)
Kargar et al.	2020	Iran	To evaluate the impact of telerehabilitation (handburn selfcare educational application) versus conventional therapy.	RCT CASP: Moderate	N=60 30 in each group; Telerehabilitation 38.2 years (+/11.7) Control 43.6 years (+/−12.6)	44 males; 16 females	Burns	Type of burn; The BurnSpecific Health Scale-Brief (BSHS-B) (includes QoL scales)	Both groups receive selfcare training during admission and at discharge provided inperson by nurse with information pamphlet; Telerehabilitation group also received instruction on the hand selfcare app which included: educational materials, opportunity to send pictures via chat and messaging system; Q&A answering; referral to clinic if needed)	Control group: nurses. App group nurses plus app clinicians plus medical professionals and ‘health care providers' (e.g., surgeons, nurses, physiotherapists, and nutrition consultants).	Author developed burns self-care app	Home	Within group, QoL, physical psychological and social dimensions and aspects of QoL improved for; both groups, QoL, physical pyshological and social dimensions and aspects of QoL significanty higher for app group
Kizony et al.	2017	Israel	To evaluate the feasibility of postdischarge 2- year hybrid synchronous - asynchronous telerehabilitation to improve upper extremity range of motion, strength, endurance, and functional ability.	Retrospective medical record audit CASP: Good	N=82 (ABI group 59.1, +/15.5, 2282 years) (MS group 57.4, +/8.2, 44–68 years)	46 males; 36 females	Acquired Brain Injury (n=74) and Multiple Sclerosis (n=8)	National Institutes of Health Stroke Scale (NIHSS); Mini-Mental State Exam (MMSE); Trail Making Test (TMT, parts A and B); Fugl- Meyer Assessment (FMA); Motor Activity Log (MAL); System Usability Scale (SUS); Focus group (on experience)	CogniMotion System which gave a hybrid synchronous - asynchronous telerehabilitation experience to improve upper extremity range of motion, strength, endurance, and functional ability	Physio or occupational therapist	CogniMotion System based program (system technical details reported in paper)	Home (with remote connectio n to clinician via call centre)	Program evaluated as usable and enjoyable; good user satisfaction; significantly improved in FMA, shoulder flexion.
Nabutovsky, Ashri et al.	2020	Israel	To evaluate the feasibility, safety, and effectiveness of a cardiac rehabilitation exercise program	Prospective observational cohort study CASP: Good	N=22 Mean age: 52.7, +/0.81	17 males; 5 females	Coronary artery disease	Smart-watch recorded minutes of aerobic exercise >70% VO2Max per week, no. resistance training sessions per week, patient questionnaires, safety = no. doctors/hospital visits, stress-test prepost, step count, no. exercise sessions >10 min per month, physiological measures, Borg Rating Perceived Exertion Scale, PHQ-9, PROMISE 10. Mobile application usage (time), remote patient management time.	Six-month secondary prevention cardiac exercise program using mobile phone applications and multidisciplinary cardiac control center services	Telerehabilitation cardiac specialist, dietitian, psychologist, exercise physiologist, physical education specialist, nurse, kinesiologist and sociologist	Datos Health mobile phone app with multidisciplinary caregiver control center/ dashboard (technical details described in paper)	Home	Patient satisfaction and app use high. Significant improve-ment in exercise capacity, functional improve-ment, and consistent aerobic program adherence. Two-thirds achieved target minutes of exercise per week; one third achieved target intensity.
PATIENT PERCEPTION STUDIES													
Source	Year	Country	Research aim	Research design [quality rating]	Sample size Age (mean, variance)	Sample Gender (male n; female n)	Sample primary Diagnosis/ Condition	Measures used in study*	Intervention (brief description)	Disciplines involved in intervention	Technologies used in delivery	Site where rehab received	Outcome
Alqahtani	2019	Saudi Arabia	To evaluate the knowledge, awareness, and perceptions of home health care patients regarding physiotherapy provided through telerehabilitation	Prospective observational cohort study CASP: Good	N=90 (males 65.8, +/ 9.4; females 58.7, +/ 7.8)	57 males; 33 females	Primary diagnosis of orthopedic problems requiring physical therapy with participant medical condition reported as orthopedic n=36; neurology n=24; sports n=15; other n=15	Telehealth Usability Questionnaire (TUQ) - translated to Arabic (awareness, knowledge, comfort); qualitative interview (on experience); interviews	Proprietary Telerehabilitat ion Technological Solutions service Telemedicine service consists of a portal to track health metrics and rehabilitation treatment plan and progress by the physical therapist, medical specialists as well as the Case Managers	Physical therapy professionals, case manager	The internet and video conferencing equipment installed at home and receiving services via video conference, including dealing with technical issues.	Home	All dimensions of the TUQ statistically improved after experience of telerehabilitation Awareness, knowledge, satisfaction increased after telerehabilitation experience
Bonnechere et al.	2017	Morocco	To evaluate the feasibility and acceptability of video games in ambulatory physical therapy.	Prospective observational cohort study CASP: Moderate	N=21 (mean reported 45 years; no variance reported)	7 males; 14 females	Tendinitis wrist/hand; Low back pain; ankylosing spondylitis, patella instability; foot fractures; balance problems; gait training; hemiparesis	Author-design questionnaire about video game exposure and access to Information technology; list of games used; extent of home use (habit); author designed survey on game acceptability; Bonnechere survey on home exercise translated to Arabic.	Physical therapy in ambulatory care with inclusion of video game instruction and use at home	Physical therapist	Mini games developed by authors for physical rehabilitation, exercise reminder using smart phone/email	Ambulatory physical therapy service department	Games were feasible in clinic setting, patients willing to try them at home, 19% afraid of falling during game, may help habit formation (only descriptive data presented)
Nabutovsky, Nachshon et al.	2020	Israel	Attitudes, perceptions, and behavioral intentions toward remote digital cardiac rehabilitation.	Cross sectional survey JBI: Good	N= 197 Mean age 64.8 years +/− 11.13 (20 to 91 years)	males; 139; 61 females	cardiac conditions	33 questions included Demographic characteristics; Lifestyle; Technological literacy and patterns of use of mobile phones, internet, computer, and monitoring devices.; Interest to receive health content through mobile phone; Interest to participate in a digital heart rehabilitation program and get telephone support	Recuperation hotel accommodati; on rehabilitation clinic - no telerehabilitati on - intervention this was exploring attritues towards potential use	No particular rehabilitation professionals identified - participant perspectives were about the use of telerehabilitation approach in general.	A range of approaches were identified and perspectives sought: cardiac-rehab telecounselling; remote digital cardiac rehab; cardiac rehab support via internet; exercise program by computer game; control over game configurations; virtual rehab class; physical activity monitoring	Rehabilitation center	Mobile phone: Text messaging was the most desired as well as email and video clips; internet; virtual reality for lifestyle managemen t, nutrition, physical activity, and mental wellbeing.
HEALTH PROFESSIONAL PERCEPTION STUDIES													
Source	Year	Country	Research aim	Research design [quality rating]	Sample size Age (mean, variance)	Sample Gender (male n; female n)	Sample primary Diagnosis/ Condition	Measures used in study*	Intervention (brief description)	Disciplines involved in intervention	Technologie s used in delivery	Site where rehab received	Outcome
Aloyuni et al.	2020	Saudi Arabia	Nationwide survey of knowledge, attitudes towards, and perceived barriers to implementing telerehabilitation in physical therapy practice; including survey instrument development of these factors	Cross sectional survey JBI: Moderate	N=347 Age not reported	106 males; 70 females	N/A	Author- developed 14 item survey - demographic information, telerehabilitation knowledge, attitudes and barriers to telerehabilitation	No - intervention this was a survey of practitioner perspectives PTs reported utilizing telerehabilitation in assessment (17%), Diagnosis (3%), Prognosis (4%), intervention (6%), and follow-up 20%)	Physiotherapists only	While 79% used no telerehabilitation a minority used magebased telerehabilitation (10%) eg. Videoconferencing; sensorbased telerehabilitation (8%) eg tilt swtiches, acceleromet ers; virtual reality telerehabilitation (3%).	Hospitals and rehabilitation centers across 13 provinces in Saudi Arabia	have %58.8 knowledge about telerehabilitation; 31.7% reported their workplaces had equipment needed; main barriers; staff skills, technical issues and cost
Ullah et al.	2020	Saudi Arabia	Nationwide survey of Knowledge, attitudes towards implementing telerehabilitation and current practice	Cross section survey JBI: Moderate	N=82 Age not reported	52 males; 30 females	N/A	14 close-ended questions targeting five domains: demographics, telemedicine knowledge, telerehabilitation service knowledge, social acceptance of these services, and risks associated with these services	No - intervention this was a survey of practitioner perspectives	Physical medicine and rehabilitation (PM&R) physicians, orthotist/ prosthetist, physiotherapists, psychologists, occupational therapists, speechlanguage pathologists and rehabilitation nurses	Access to the following: smart phone/ simple phone	Primary, secondary, tertiary hospitals	Most participants think telerehabilitation is important, but most are not currently involved. There is a need for telerehabilitation guidelines and addressing the barriers pertaining to training, resources, cost, policy making, confidentiality, and perception of patients

To provide a narrative synthesis of findings, the text content of each source was considered using thematic analysis. NVivo 12 plus™ software (QSR International, 2020) was used to extract text data and provide the platform for content analysis to create themes and subthemes closely aligned with the data. A consensus approach was taken by the research team to enable theme development using keywords and phrases informed by literature reviewed in the introduction.

## RESULTS

Twelve articles published in English met the inclusion criteria. The articles' years of publication ranged from 2015 to 2020 (mode 2020, 2018). Four of 19 MENA countries produced research: Iran (n=2), Israel (n=5), Morocco (n=1), and Saudi Arabia (n=4). There were 10 studies involving patients and two sampled health professionals. There were four randomised controlled trials and five cohort studies (four prospective, one retrospective). Three studies employed cross-sectional surveys. Methodological quality was rated moderate-to-good for all studies.

Patient studies were conducted in Israel (n=5), Iran (n=2), Morocco (n=1) and Saudi Arabia (n=2). A total of 621 patients were involved (sample size ranged from 12 to 200; mean 62.1). All patients were community-dwelling adults, with an age range of 18 to 85 years. Most required physical rehabilitation for musculoskeletal disorders, burns, and cardiovascular conditions (excluding stroke) (Table 3). One study was about neurorehabilitation with participants having conditions including acquired brain injury and multiple sclerosis (Kizony et al., 2017).

Seven studies examined intervention impact (Table 3). Interventions were provided by occupational therapists (Golebowicz et al., 2015), physiotherapists (Alasfour & Almarwani, 2020; Kalron et al., 2018), occupational therapists and/or physiotherapists (Kizony et al., 2017), rehabilitation physician (Azma et al., 2018) and multidisciplinary rehabilitation teams (Kargar et al., 2020; Nabutovsky, Ashri et al., 2020). Rehabilitation program goals were predominantly directed toward decreasing impairment (Alasfour & Almarwani, 2020; Azma et al., 2018; Golebowicz et al., 2015; Kalron et al., 2018; Kizony et al., 2017; Nabutovsky, Ashri et al., 2020b) or enhancing quality of life (Kargar et al., 2020). Telerehabilitation delivery modalities included: computer-based software (Golebowicz et al., 2015; Kalron et al., 2018; Kizony et al., 2017), phone (Azma et al., 2018), and phone ‘apps’ (Alasfour & Almarwani, 2020; Kargar et al., 2020; Nabutovsky, Ashri et al., 2020b).

Three studies explored patient perceptions of telerehabilitation (Alqahtani, 2019; Bonnechere et al., 2017; Nabutovsky, Nachschon et al., 2020) and two investigated the perceptions of rehabilitation professionals (Aloyuni et al., 2020; Ullah et al., 2020). The patient studies used different settings. In Saudi Arabia patients came from home health care settings where physiotherapy was delivered using telerehabilitation (Alqahtani, 2019). In Morocco there was clinic and at home use of online game technology for physiotherapy exercise practice (Bonnechere et al., 2017), and in Israel post-hip surgery telerehabilitation was conducted at home (Kalron et al., 2018). Professionals who shared perceptions all came from Saudi Arabia. In one study (Aloyuni et al., 2020), a national survey of physiotherapists revealed attitudes towards telerehabilitation practice. In the other study a multidisciplinary sample of rehabilitation professionals from physical medicine and rehabilitation doctors, orthotist/prosthetist, physiotherapists, psychologists, occupational therapists, speech-language pathologists, and rehabilitation nurses described their knowledge, acceptance, use and perceived risks of telerehabilitation (Ullah et al., 2020).

All intervention studies and studies about patient perceptions used standardized instruments to collect data on one or more variables; the names of the instruments are listed in Table 3. There was no consistency in instrumentation use because each study aimed to answer a different intervention question. The two professional perception studies used author-designed self-report surveys. The only standardized instrument that was specifically designed to ascertain views about the delivery of service by information technologies was the Telehealth Usability Questionnaire, (TUQ) developed by Parmanto et al. (2016), cited by Langbecker et al. (2017) and used in the Alqahtani (2019) study.

While an inspection of Table 3 reveals all telerehabilitation interventions achieved statistically significant improvements in primary outcomes, the intervention questions, study designs and impact measures were too variable to permit a meta-analysis of findings.

Narrative synthesis was performed by analysing article content and identifying common concept categories, which were then iteratively grouped first into subthemes and then into four themes. All 12 sources were included in this synthesis. Four themes were evident: (1) rehabilitation availability and accessibility, (2) perceptions of telerehabilitation, (3) telerehabilitation to augment traditional rehabilitation services, and (4) barriers to telerehabilitation. Each of these is now described.

### THEME 1: REHABILITATION AVAILABILITY AND ACCESSIBILITY

This theme captures the remarkable lack of, or restricted access to rehabilitation services in MENA. The limited access to rehabilitative care services was particularly evident in remote areas (Bonnechere et al., 2017; Ullah et al., 2020). Services were more available in urban areas than in rural areas (Aloyuni et al., 2020; Ullah et al., 2020). Some participants had to travel to obtain the rehabilitation services they required in major cities (Aloyuni et al., 2020; Ullah et al., 2020). Due to the COVID-19 outbreak, access to rehabilitation services were restricted (Aloyuni et al., 2020). A shortage of rehabilitation professionals was also identified (Bonnechere et al., 2017; Ullah et al., 2020).

There were obstacles to rehabilitation service availability and access. After completion of a patient's hospital healthcare and their discharge, there was a lack of continuity of rehabilitative care due to non-adherence and a lack of community-based rehabilitation programs (Ullah et al., 2020). Poor adherence to traditional rehabilitative services among patients occurred due to distance from rehabilitation services, job demands (Golebowicz et al., 2015; Kalron et al., 2018; Nabutovsky, Ashri et al., 2020), time constraints, transportation issues (Alasfour & Almarwani, 2020; Alqahtani, 2019; Nabutovsky, Ashri et al., 2020), family/household responsibilities, commitments and social duties (Alasfour & Almarwani, 2020; Alqahtani, 2019), and personal beliefs and habits (Bonnechere et al., 2017).

### THEME 2: PERCEPTIONS OF TELEREHABILITATION

This theme revealed perceptions of patients and practitioners about using various forms of telerehabilitation. Patient-participants in a telerehabilitation intervention study indicated they were satisfied with and comfortable using telerehabilitation software technologies (Alqahtani, 2019; Bonnechere et al., 2017; Kizony et al., 2017).

In one cross-sectional study, most patients were willing to undergo rehabilitation follow-ups and therapeutic communication by phone (Nabutovsky, Nachschon et al., 2020a). The phone is a frequently used method for telerehabilitation; is user-friendly due to the variety of call features or apps (Alasfour & Almarwani, 2020; Azma et al., 2018; Kargar et al., 2020; Nabutovsky, Ashri et al., 2020); and is also attractive to more elderly patients (Azma et al., 2018).

Studies by Aloyuni et al (2020) and Ullah et al (2020) reported that the majority of rehabilitation professionals regarded telerehabilitation as a valuable way to deliver rehabilitation services despite the fact that most of them did not use it in their practice.

### THEME 3: TELEREHABILITATION TO AUGMENT TRADITIONAL REHABILITATION SERVICES

Across the studies, telerehabilitation was reported as a service provided in addition to typical rehabilitation care. It was reported as having effective results as compared to conventional care sessions (Alasfour & Almarwani, 2020; Kalron et al., 2018; Kargar et al., 2020). Telerehabilitation was also suggested to have the potential to decrease hospital admissions and length of hospital stay (Azma et al., 2018; Kalron et al., 2018).

Telerehabilitation enabled continuing rehabilitation care after hospital discharge and follow-up (Alasfour & Almarwani, 2020; Kalron et al., 2018; Kizony et al., 2017; Kargar et al., 2020; Ullah et al., 2020), which improved quality of life and outcomes (Kargar et al., 2020; Ullah et al., 2020). It also increased engagement with rehabilitation services remotely compared to traditional services (Alasfour & Almarwani, 2020; Kalron et al., 2018) and demonstrated good adherence (Nabutovsky, Ashri et al., 2020).

Participation rate in rehabilitation services was good with telerehabilitation. Telerehabilitation gave an alternative option to hospital or clinic-based service (Nabutovsky, Ashri et al., 2020) and permitted collaboration with rehabilitation professionals (Kizony et al., 2017), therapeutic relationships (Kargar et al., 2020) and person-centered care (Nabutovsky, Nachschon et al., 2020). Telerehabilitation services attracted good patient satisfaction (Nabutovsky, Ashri et al., 2020) and remote sessions with the same therapist who provides the in-person sessions increases patient satisfaction and confidence (Alqahtani, 2019).

Telerehabilitation helped to facilitate remote access to rehabilitation services (Alasfour & Almarwani, 2020; Alqahtani, 2019; Aloyuni et al., 2020; Azma et al., 2018; Bonnechere et al., 2017; Golebowicz et al, 2015; Kalron et al., 2018; Nabutovsky, Ashri et al., 2020), especially for patients who could or would not attend in-person rehabilitation sessions (Alasfour & Almarwani, 2020; Azma et al., 2018; Alqahtani, 2019; Nabutovsky, Nachschon et al., 2020), and/or whose physical presence in the clinic was not required (Kalron et al., 2018).

Due to the COVID-19 lockdown and the required social distancing, telerehabilitation was identified to be the best approach to deliver rehabilitation services and avoid infection (Aloyuni et al., 2020).

Telerehabilitation helped to reduce treatment expenses compared to traditional rehabilitation (Azma et al., 2018; Golebowicz et al., 2015; Kargar et al., 2020). The use of telerehabilitation saved time and reduced absences from work for patients (Alasfour & Almarwani, 2020; Alqahtani, 2019; Azma et al., 2018; Golebowicz et al., 2015; Nabutovsky, Ashri et al., 2020), and their supporting relatives (Alqahtani, 2019).

Finally, telerehabilitation use addressed the shortage of providers and inadequate public health service infrastructure (Bonnechere et al., 2017; Golebowicz et al, 2015).

### THEME 4: BARRIERS TO TELEREHABILITATION

This theme identified barriers to the implementation of telerehabilitation, specifically the difficulties of capacity in infrastructure, policies, guidelines, and practitioner expertise. Infrastructure and resourcing issues presented a major barrier. These included ways in which standards and processes for information and communication technologies (ICT) and engaging service providers/users from the initial implementation stages of a telehealth project could directly impact the integration of telerehabilitation services into practice. Consequently, the barriers involved a lack of funding and investments into infrastructure; the high cost of ICT; the rapidly changing nature of ICT; and ICT innovation needs, such as the availability of suitable devices and equipment, internet speed, and usability (Aloyuni et al., 2020; Bonnechere et al., 2017; Ullah et al., 2020).

The lack of telerehabilitation privacy policies, standards, guidelines, and data protection regulations was found to create risks such as compromised patient data security and patient privacy, and consultations with unauthorised persons (Ullah et al., 2020).

The lack of collaboration between educational and governmental authorities in establishing local telerehabilitation guidelines was another barrier. Such guidelines must be fully compatible with local conditions, such as culture and language, local telerehabilitation scenarios, the rehabilitation strategies of the local teams, and localized functional evaluation of the patients (Bonnechere et al., 2017; Ullah et al., 2020).

Barriers also included challenges related to human capacity-building in the development of knowledge, skills, and attitude for both providers and patients. There was a lack of awareness and knowledge about telerehabilitation technologies and applications among patients and rehabilitation professionals (Alqahtani, 2019; Aloyuni et al., 2020; Ullah et al., 2020).

Telerehabilitation training is required for rehabilitation providers and patients to use the relevant technology, and continuous support will also increase their confidence (Alqahtani, 2019; Azma et al., 2018; Ullah et al., 2020). It was reported that the patients' confidence in using the telerehabilitation platform increased after their first experience (Alqahtani, 2019). There was a lack of expertise regarding telerehabilitation adoption of technology and a shortage of human capital (Aloyuni et al., 2020). A lack of acceptance among clinicians is one likely reason for the low uptake and maintenance of telerehabilitation (Alqahtani, 2019; Ullah et al., 2020). Policymakers' attitudes also obstructed the use of telerehabilitation services (Aloyuni et al., 2020; Ullah et al., 2020).

The cultural and social context was considered a barrier limiting telerehabilitation implementation (Bonnechere et al., 2017; Ullah et al., 2020); for example, patients undertook physical rehabilitation activities fully clothed (Bonnechere et al., 2017).

## DISCUSSION

This structured review aimed to characterize and summarize information published in peer-reviewed journals about telerehabilitation practice in MENA. All included sources were recent (i.e., in the past five years). This aligns with previous WHO reports indicating telehealth practice was increasing in this region. The upward trajectory of publication numbers indicates more research evidence will emerge in years to come. Currently, telerehabilitation studies were limited to only a quarter of all MENA countries; 15 MENA countries had no sources identified. It is hoped that evidence will emerge from as yet unrepresented MENA countries, so in time a more complete picture of telerehabilitation in this region can be made. No systematic review could be located and the present study did not have the data necessary for a meta-analysis. However, this study provides the only current summary and synthesis of research evidence on telerehabilitation in this region or from/about any country within the MENA region.

Research studies used RCT, cohort, and cross-sectional study designs, a variety of outcome measures and were of moderate to good methodological quality. Studies were either evaluations of telerehabilitation interventions (n=7) conducted in homes or in the workplace using a variety of technologies and platforms, or they were studies about practitioner or patient perceptions of current/future telerehabilitation (n=5). Interventions all produced positive significant outcomes. In intervention studies, health conditions of patient participants were cardiovascular, musculoskeletal, neurological or burns. Only one of these conditions, cardiovascular, is a major cause of MENA mortality; non-communicable disease (NCD) accounts for 74% of all deaths in MENA (Kaneda & El-Saharty, 2017) and the four most common MENA NCDs are cardiovascular, diabetes, cancer, and chronic respiratory disease. Each of these NCDs produce impairment, activity limitation and participation restrictions that could benefit from rehabilitation interventions. Future research could explore use of telerehabilitation in management of the consequences of these other common NCDs.

Rehabilitation intervention professionals involved were occupational therapists, physical therapists, rehabilitation doctors or, in two papers, “multidisciplinary rehabilitation teams” with broad engagement. Involvement of these disciplines in telerehabilitation is consistent with international professional practice standards (e.g., World Federation of Occupational Therapists, 2021; World Physiotherapy, 2019). Studies exploring professionals' perceptions about the current or future use of telerehabilitation included disciplines beyond the three named disciplines in intervention studies; but most evidence about professionals' perceptions came from physical therapists in Saudi Arabia. Future research should explore perceptions of health professionals from different disciplines, from those who work in teams versus those who work without collaboration, and from different countries in MENA. Such information could help build an understanding of the appetite and readiness for telerehabilitation practice in addition to capacity and development needs.

Narrative synthesis of content revealed four themes. The first was “rehabilitation availability and accessibility.” The challenges of insufficient provision of rehabilitation services, concentration of services in major metropolitan settings and lack of community-based rehabilitation aligned with WHO reports of rehabilitation service challenges in the MENA region (WHO, 2011, 2019, 2020).

The second theme was “perceptions of telerehabilitation.” Here, patients perceived telerehabilitation to be user-friendly and an acceptable approach for intervention or follow up with good patient satisfaction. This reflects similar positive patient views about usability, acceptability, and satisfaction revealed in a recent global systematic review of stakeholder adoption of telerehabilitation services (Niknejad Ismail, Bahari & Nazari, 2021). Professionals were also positive in their perceptions about the value of telerehabilitation even though most had not used it. This finding suggests more research is needed regarding rehabilitation professionals' acceptance of and readiness to use technology for rehabilitation service delivery in practice - a conclusion also drawn by Niknejad et al (2021).

The third theme was “telerehabilitation to augment traditional rehabilitation services.” In this theme, the weight of evidence tended toward telerehabilitation as complementing rather than replacing traditional in-person rehabilitation services (e.g., as follow-up or as an additional service) (Laver et al., 2020). This was also reported in the systematic review by Niknejad et al (2021). That study also found concerns by health professionals about the increased work responsibilities that come from use of technology-enabled telerehabilitation and perceived risks to professional status. Further research about these and other issues relating to telerehabilitation in MENA as an augmentative or alternative to in-person service is worth investigation.

The fourth and final theme, “barriers to telerehabilitation” covered resource, training and capacity issues. Some of these have previously been identified in literature, as examples: insufficient hardware and software, low connectivity and reliance on decisions made by management (Jafni, Bahari, Ismail & Hanafi, 2019). Barriers to telerehabilitation revealed in this review were:

- Systematic barriers relating to rehabilitation workforce shortages in general (Bonnechere et al., 2017; Ullah et al., 2020), and reliance on rehabilitation administrators' decisions regarding practice delivery and resources (Aloyuni et al., 2020; Ullah et al., 2020).
- Information technology infrastructure limitations in the health system and the economy in general (Aloyuni et al., 2020; Bonnechere et al., 2017; Golebowicz et al., 2015; Ullah et al., 2020) and a reliance on mobile phones (Alasfour & Almarwani, 2020; Azma et al., 2018; Kargar et al., 2020; Nabutovsky, Ashri et al., 2020; Nabutovsky, Nachschon et al., 2020).
- Lack of awareness, acceptance, confidence, expertise and implementation-ready staff and patients (Alqahtani, 2019; Azma et al., 2018; Aloyuni et al., 2020; Ullah et al., 2020), and
- Lack of guidelines and policy arrangements to support confidentiality and security of technology-enabled rehabilitation in ways that not only protect patients and staff but are also appropriate to cultural and religious norms and standards specifically related to MENA (Bonnechere et al., 2017; Ullah et al., 2020).

Despite these barriers, the review also identified a number of enablers of uptake:

- Widespread availability and use of smart-phone features and the acceptability and utility of the phone as a platform for rehabilitation interventions (Alasfour & Almarwani, 2020; Azma et al., 2018; Kargar et al., 2020; Nabutovsky, Ashri et al., 2020; Nabutovsky, Nachschon et al., 2020).
- Positive attitudes of rehabilitation professionals and patients to the idea of telerehabilitation and willingness to participate (Alqahtani, 2019; Bonnechere et al., 2017; Nabutovsky, Nachschon et al., 2020a; Ullah et al., 2020).
- Successful examples of telerehabilitation implementation with a range of diagnostic groups, with different rehabilitation personnel as providers from distance (Alasfour & Almarwani, 2020; Azma et al., 2018; Golebowicz et al., 2015; Kalron et al., 2018; Kargar et al., 2020; Kizony et al., 2017; Nabutovsky, Ashri et al., 2020).
- Increased program adherence and participation observed with telerehabilitation (Alasfour & Almarwani, 2020; Kalron et al., 2018; Nabutovsky, Ashri et al., 2020a).
- Ease of participation for patients particularly in relation to reduced work absence and saving time (Alasfour & Almarwani, 2020; Azma et al., 2018; Golebowicz et al., 2015; Nabutovsky, Ashri et al., 2020).
- Use of telerehabilitation technologies in treatment training increased patients' confidence and comfortability (Alqahtani, 2019; Bonnechere et al., 2017), and
- Noticeable health outcomes for patients in telerehabilitation programs (Alasfour & Almarwani, 2020; Kalron et al., 2018; Kargar et al., 2020).

## LIMITATIONS

This review used a structured, replicable approach across multiple databases to summarize and appraise sources relevant to telerehabilitation in the MENA region. There were limitations in the design of the study. The search ended March 2021. Only peer-reviewed published sources in English were included, even though one of the investigators was fluent in Arabic. While this limited the number of research studies included in the analysis, other sources in Arabic or that did not meet inclusion criteria but were relevant to the topic, were included in the introduction section of this paper, for example, Nafai et al., (2017) and Qureshi et al., (2021). The decision to include English-only sources was made to ensure replicability of the search strategy to enhance the methodological strength of the review. It was noteworthy that very few Arabic-language sources were, in any event, found.

## CONCLUSION

Telerehabilitation is an emerging practice in MENA. Occupational therapists, physiotherapists and rehabilitation doctors are most commonly involved. The limited research evidence available suggests that telerehabilitation is implemented predominantly in the home for patient health conditions and technology platforms that reflect practitioner or provider specialisation. Only one of the top four NCD mortality related conditions in MENA have evidence of telerehabilitation use. Interventions achieve statistically significant outcomes and patients are generally satisfied about their experience or positive about the idea of telerehabilitation. Rehabilitation professionals too are positive, but as yet most have scant experience and there are general rehabilitation workforce shortages with services centralised and a lack of community-based programs that create existing pressures on practitioners.

The research evidence in this review suggests implementation of telerehabilitation in MENA is feasible, acceptable to patients, and relevant to a range of professions and health conditions. The challenge is not whether telerehabilitation can be done in MENA, but how it can be done at scale so that more rehabilitation services are available, and patients have more efficient access to services.

Implementation and uptake of telerehabilitation requires readiness in regulatory and policy systems, capacity and security in technological infrastructure, confidence and competence of rehabilitation professionals and acceptance by patients and their families of this method of service delivery. While this review provides a starting point for an evidence-based approach to understanding telerehabilitation in MENA, continued research effort is required to support managers and policy makers, practitioners, and patients to consider when, how, and where to adopt this approach to service delivery and ensure its relevance and effectiveness.

## APPENDIX MEDLINE VIA (OVID INTERFACE): *RESULT (N= 6)*

1. telehealth.mp. or Telemedicine/
2. tele-health.mp.
3. telemedicine.mp.
4. tele-medicine.mp.
5. telerehabilitation.mp. or Telerehabilitation/
6. tele-rehabilitation.mp.
7. ehealth.mp.
8. e-health.mp.
9. rehabilitation.mp. or Rehabilitation/
10. habilitation.mp.
11. middle east/ or bahrain/ or iran/ or iraq/ or israel/ or jordan/ or kuwait/ or lebanon/ or oman/ or qatar/ or saudi arabia/ or syria/ or united arab emirates/ or yemen/ (102498)
12. Palestine.mp.
13. africa, northern/ or algeria/ or egypt/ or libya/ or morocco/ or tunisia/ or mauritania/
14. “western sahara”.mp.
15. “mobile health”.mp.
16. mhealth.mp.
17. 1 or 2 or 3 or 4 or 5 or 6 or 7 or 8 or 15 or 16
18. 9 or 10
19. 11 or 12 or 13 or 14
20. 17 and 18 and 19
21. limit 20 to yr=“1990 - 2020”

Allied and Complementary Medicine Database (AMED): *Result (N=2),* Excerpta Medica Database (EMBASE): *Result (N=24),* Joanna Briggs Institute (JBI) EBP Database: *Result (N= 0)* AND The Cochrane Database of Systematic Reviews (CDSR): *Result (N=0); Same as Medline above via (OVID interface).*

Scopus: a comprehensive database for scientific, technical and medical information (Scopus web interface): *Result (N=36)*

( ( ( TITLE-ABS-KEY ( telehealth ) OR TITLE-ABS-KEY ( telehealth ) ) AND PUBYEAR > 1989 AND PUBYEAR < 2021 ) OR ( ( TITLE-ABS-KEY ( telemedicine ) OR TITLE-ABS-KEY ( tele-medicine ) ) AND PUBYEAR > 1989 AND PUBYEAR < 2021 ) OR ( ( TITLE-ABS-KEY ( telerehabilitation ) OR TITLE-ABS-KEY ( telerehabilitation ) ) AND PUBYEAR > 1989 AND PUBYEAR < 2021 ) OR ( ( TITLE-ABS-KEY ( ehealth ) OR TITLE-ABS-KEY ( e-health ) ) AND PUBYEAR > 1989 AND PUBYEAR < 2021 ) ) OR ( ( TITLE-ABS-KEY ( “mobile health” ) OR TITLE-ABS-KEY ( mhealth ) ) AND PUBYEAR > 1989 AND PUBYEAR < 2021 ) )

AND ( ( TITLE-ABS-KEY ( rehabilitation ) OR TITLE-ABS-KEY ( habilitation ) ) AND PUBYEAR > 1989 AND PUBYEAR < 2021 )

AND ( ( ( TITLE-ABS-KEY ( “middle east” OR bahrain OR qatar OR “United Arab Emirates” OR yemen OR iran OR iraq OR israel ) OR TITLE-ABS-KEY ( jordan OR kuwait OR lebanon OR oman OR syria OR “Saudi Arabia” OR palestine ) ) AND PUBYEAR > 1989 AND PUBYEAR < 2021 ) OR ( TITLE-ABS-KEY ( “North Africa” OR tunisia OR egypt OR morocco OR “Western Sahara” OR algeria OR libya OR mauritania ) AND PUBYEAR > 1989 AND PUBYEAR < 2021 ) )

CINAHL: Cumulative Index to Nursing & Allied Health Literature via (EbscoHost): *Result (N=17)*

S1. (MH “Telehealth+”) OR “telehealth OR tele-health”

S2. (MH “Telemedicine+”) OR “telemedicine”

S3. “tele-medicine”

S4. (MH “Telerehabilitation”)

S5. “tele-rehabilitation”

S6. “ehealth”

S7. “e-health”

S8. ““mobile health””

S9. “mhealth”

S10. S1 OR S2 OR S3 OR S4 OR S5 OR S6 OR S7 OR S8 OR S9

S11. (MH “Rehabilitation+”) OR “rehabilitation”

S12. “habilitation”

S13. S11 OR S12

S14. (MH “Yemen”) OR (MH “United Arab Emirates”) OR (MH “Syria”) OR (MH “Saudi Arabia”) OR (MH “Qatar') OR (MH “Oman”) OR (MH “Lebanon”) OR (MH “Kuwait”) OR (MH “Jordan”) OR (MH “Israel”) OR (MH “Iraq”) OR (MH “Iran”) OR (MH “Bahrain”) OR (MH “Middle East+”)

S15. “palestine”

S 16. (MH “Africa, Northern+”) OR (MH “Algeria”) OR (MH “Egypt”) OR (MH “Libya”) OR (MH “Morocco”) OR (MH “Tunisia”) OR (MH “Mauritania”) OR “( “north africa” OR Tunisia OR Egypt OR Morocco ) OR ( Algeria OR Libya OR Mauritania OR “western Sahara” )”

S 17. S14 OR S15 OR S16

S 18. S10 AND S13 AND S17

S 19. S10 AND S13 AND S17 (Limiters - Published Date: 19900101-20201231) **Web of Sciences (WoS): *Result (N= 20)***

**# 1 TOPIC:** (telehealth) *OR* **TOPIC:** (tele-health)

*Indexes=SCI-EXPANDED, SSCI, A&HCI, CPCI-S, CPCI-SSH, ESCI, CCR-EXPANDED, IC Timespan*

**# 2 TOPIC:** (telemedicine) *OR* **TOPIC:** (tele-medicine)

*Indexes=SCI-EXPANDED, SSCI, A&HCI, CPCI-S, CPCI-SSH, ESCI, CCR-EXPANDED, IC Timespan =1990-2020 =1990-2020*

**# 3 TOPIC:** (telerehabilitation) *OR* **TOPIC:** (tele-rehabilitation)

*Indexes=SCI-EXPANDED, SSCI, A&HCI, CPCI-S, CPCI-SSH, ESCI, CCR-EXPANDED, IC Timespan=1990-2020*

**# 4 TOPIC:** (ehealth) *OR* **TOPIC:** (e-health)

*Indexes=SCI-EXPANDED, SSCI, A&HCI, CPCI-S, CPCI-SSH, ESCI, CCR-EXPANDED, IC Timespan=1990-2020*

**# 5 TOPIC:** (“mobile health”) *OR* **TOPIC:** (mhealth)

*Indexes=SCI-EXPANDED, SSCI, A&HCI, CPCI-S, CPCI-SSH, ESCI, CCR-EXPANDED, IC Timespan=1990-2020*

**# 6 TOPIC:** (“Middle East” OR Bahrain OR Qatar OR “United Arab Emirates” OR Yemen) *OR* **TOPIC:** (Iran OR Iraq OR Israel OR Jordan OR Kuwait OR Lebanon) *OR* **TOPIC:** (Oman OR Syria OR “Saudi Arabia” OR Palestine)

*Indexes=SCI-EXPANDED, SSCI, A&HCI, CPCI-S, CPCI-SSH, ESCI, CCR-EXPANDED, IC Timespan=1990-2020*

**# 7 TOPIC:** (“North Africa” OR Tunisia OR Egypt OR Morocco OR “Western Sahara”) *OR* **TOPIC:** (Algeria OR Libya OR Mauritania)

*Indexes=SCI-EXPANDED, SSCI, A&HCI, CPCI-S, CPCI-SSH, ESCI, CCR-EXPANDED, IC Timespan=1990-2020*

**# 8 TOPIC:** (rehabilitation) *OR* **TOPIC:** (habilitation)

*Indexes=SCI-EXPANDED, SSCI, A&HCI, CPCI-S, CPCI-SSH, ESCI, CCR-EXPANDED, IC Timespan=1990-2020*

**# 9** #5 OR #4 OR #3 OR #2 OR #1

*Indexes=SCI-EXPANDED, SSCI, A&HCI, CPCI-S, CPCI-SSH, ESCI, CCR-EXPANDED, IC Timespan=1990-2020*

**# 10** #7 OR #6

*Indexes=SCI-EXPANDED, SSCI, A&HCI, CPCI-S, CPCI-SSH, ESCI, CCR-EXPANDED, IC Timespan=1990-2020*

**# 11** #10 AND #9 AND #8

*Indexes=SCI-EXPANDED, SSCI, A&HCI, CPCI-S, CPCI-SSH, ESCI, CCR-EXPANDED, IC Timespan=1990-2020*
